# Development and properties of genetically encoded pH sensors in plants

**DOI:** 10.3389/fpls.2013.00523

**Published:** 2013-12-18

**Authors:** Alexandre Martinière, Guilhem Desbrosses, Hervé Sentenac, Nadine Paris

**Affiliations:** ^1^Biochimie et Physiologie Moléculaire des Plantes, Institut de Biologie Intégrative des Plantes, UMR 5004 CNRS/UMR 0386 INRA/Montpellier SupAgro/Université Montpellier 2Montpellier, France; ^2^Laboratory of Tropical and Mediterranean Symbioses (UMR113, Université Montpellier 2, Institut de Recherche pour le Développement, Cirad Montpellier SupAgro, Institut National de la Recherche Agronomique), Université Montpellier 2Montpellier, France

**Keywords:** pH, GFP, vacuoles, pHluorin, acidic gowth theory, ion transport, endomembrane trafficking

## Abstract

Fluorescent proteins (FPs) have given access to a large choice of live imaging techniques and have thereby profoundly modified our view of plant cells. Together with technological improvements in imaging, they have opened the possibility to monitor physico-chemical changes within cells. For this purpose, a new generation of FPs has been engineered. For instance, pHluorin, a point mutated version of green fluorescent protein, allows to get local pH estimates. In this paper, we will describe how genetically encoded sensors can be used to measure pH in the microenvironment of living tissues and subsequently discuss the role of pH in (i) exocytosis, (ii) ion uptake by plant roots, (iii) cell growth, and (iv) protein trafficking.

## INTRODUCTION

Genetically encoded fluorescent sensors are becoming extremely powerful tools for functional imaging. For instance, the classic calcium sensor introduced by Roger Tsien’s lab, cameleon, is now extensively used in the plant field (Miyawaki et al., 1997; Romoser et al., 1997). Such calcium sensors have allowed major breakthroughs regarding, for instance, the molecular dialog between plant roots and symbiotic microorganisms (Sieberer et al., 2012; Singh and Parniske, 2012). More recently, dedicated strategies have led to the development of very promising biosensors, which allow to probe the local concentration of disaccharides, phosphate, amino acid, or ammonium (reviewed in Gjetting et al., 2013; Jones et al., 2013). In this perspective issue, we will focus on a specific type of sensors optimized for pH measurement.

## PROPERTIES OF GENETICALLY ENCODED pH SENSORS

Classical fluorescent dyes have been used to estimate pH in plants. For example, fluorescein coupled to dextran allows pH recording in the close vicinity of roots (Monshausen et al., 2009, 2011) or shoots (Geilfus and Mühling, 2012). The 2′,7′-Bis(2-carboxyethyl)-5(6)-carboxyfluorescein (BCECF), which was first introduced by Rink et al. (1982), is also classically used to measure plant vacuolar pH, especially in its membrane permeable form as acetomethyl ester (BCECF-AM; Krebs et al., 2010). Nevertheless, such approaches are invasive and do not allow measuring pH in other intracellular cell structures. Genetically encoded pH sensors have been designed to overcome these limitations.

The chromophore of green fluorescent protein (GFP) is formed by two autocatalytic reactions (cyclization and oxidation) between Ser65, Tyr66, and Gly67 (review in Wachter, 2007). It absorbs light at two maxima: 395 nm when protonated and 475 nm when depronated (Bizzarri et al., 2009). This shift in absorption has been extensively used to record pH in living organisms (Kneen et al., 1998; Llopis et al., 1998), and optimized versions of GFP for pH measurement have been generated. Ecliptic and superecliptic pHluorins display almost no fluorescence when protonated, allowing sensitive detection of biological processes associated with pH increase (**Table 1**; Miesenböck et al., 1998; Sankaranarayanan and Ryan, 2001). However, the equilibrium between the protonated and deprotonated states of these proteins is also affected by temperature and ionic strength. Furthermore, the light emitted by such sensor protein is dependent on its own concentration. Thus a variation in fluorescence signal can result either from a change in pH or in concentration of the sensor. To circumvent this issue, ratiometric pH sensors have been developed providing a pH readout independent of the probe concentration. Among such sensors, one of the most popular is pHluorin. It was generated by introducing point mutations in *Aequorea victoria* GFP (AvGFP) sequence, especially S202H, which conferred ratiometric behavior to the sensor (Miesenböck et al., 1998; Bizzarri et al., 2009). For example, pHluorin displays a strong increase in absorption at 475 nm and, simultaneously, a strong decrease in absorption at 395 nm when the pH is shifted from 7.5 to 5.5. The pHluorin sensor has a pKa of 6.9 and the dynamic range of fluorescent signal is from pH 5.4 to 8.4 (**Table 1**). This pH range is suitable for most plant subcellular compartments, with the exception of the apoplast, which is too acidic, and the vacuole, where GFP is degraded (Tamura et al., 2003). Later, derivatives of this protein were engineered to improve the level of expression. This is the case for ratiometric Venus Citrine (RaVC), whose codon usage has been optimized for expression in *Aspergillus* (Bagar et al., 2009), and the plant-solubility-modified ratiometric pHluorin (*PRpHluorin*), optimized for *Arabidopsis* (Shen et al., 2013). To avoid erroneous pH measurement due to chromatic aberrations, an alternative type of ratiometric sensor was generated from S65T GFP, where the emission is yellow-shifted upon alkalization (**Table 1**). This dual emission GFP (deGFP) contains the point mutations T203C and H148G or H148C (Hanson et al., 2002). In addition, some sensors such as E^2^GFP and E^1^GFP combine dual emission and dual excitation features (Bizzarri et al., 2006; Arosio et al., 2007).

**Table 1:** Selected genetically encoded pH sensors available for *in vivo* pH measurements.

Constructs	Type	Excitation (nm)	Emission (nm)	pH range	pKa	Delta ratio	Reference
pHluorin, pHluorin2^*^, RaVC^**^, PRpHluorin^***^, pHGFP^****^	Ratiometric	395/475	510	5.4–8.4	6.9	2.5	Miesenböck et al. (1998)
Ecliptic pHluorin, PEpHluorin^***^	Ecliptic	395/477	510	6.5–8	7.2	13.5	Miesenböck et al. (1998)
Superecliptic pHluorin	Ecliptic	477	510	5.5–8.5	7.2	13	Sankaranarayanan and Ryan (2001)
deGFP3	Ratiometric	396	460/515	6–9	7.3	20	Hanson et al. (2002)
E2GFP	Ratiometric	458/488	500/560	5–8.5	6.9/7.5	10	Bizzarri et al. (2006)
ptGFP	Ratiometric	390/475	540	3.8–8.2	7.3	36	Schulte et al. (2006)
pHlameleon 6	FRET-based ratiometric	458	481/533	5–7.5	5.6	9	Esposito et al. (2008)
pHusion	Ratiometric	488/558	510/600	4.5–8	5.8	6	Gjetting et al. (2012)
pHlash	BRET	n.a.	475/525	5.4–9	n.a.	3.3	Zhang et al. (2012)

* Eightfold brighter version of pHluorin (Mahon, 2011)

** pHluorin with optimize codon usage for *Aspergillus* (Bagar et al., 2009)

*** pHluorin with optimize codon usage for *Arabidopsis* (Shen et al., 2013)

**** pHGFP, chimera between smGFP (Davis and Vierstra, 1998) and pHluorin to prevent splicing (Moseyko and Feldman, 2001)

The major limitation of using pHluorin and its derivates, especially in plant tissues, is their acidic quenching at an apparent pH near 4.5. Two strategies were used to improve pH sensing in acidic environments. The first strategy is to use a fluorescent protein (FP), *Pt-*GFP, from orange sea pen, *Ptilosarcus gurneyi* (Schulte et al., 2006). Although *Pt*-GFP has a pKa similar to that of pHluorin (pK_PtGFP_: 7.3), this protein has a broader pH response range and is more stable in acidic conditions (Schulte et al., 2006). The second strategy is to make a tandem of FPs with highly different ranges of pH responsiveness. The measure of pH is then either given by Förster resonance energy transfer (FRET) between these two proteins, as for pHlameleon (Esposito et al., 2008) or by the ratio of fluorescence of the proteins. In particular, the tandem of enhanced GFP (eGFP) and monomeric red FP (mRFP1) is suitable for measuring pH in the apoplasm (Gjetting et al., 2012). Finally, a new pH biosensor was developed recently based on bioluminescence resonance energy transfer (BRET). This pHlash sensor is composed of a luciferase as a donor, fused to pH-sensitive Venus as acceptor (Zhang et al., 2012). Use of bioluminescence significantly improves the signal-to-noise ratio making BRET particularly useful for experimentation at the whole tissue or organism scale.

Regardless of the exact type of genetically encoded pH sensors, estimation of the local pH value from the fluorescence data is not straightforward. This requires calibration to characterize the relationship between the fluorescence properties of the sensor and pH. The calibration can be carried out either *in vitro* or *in vivo* (Gao et al., 2004; Schulte et al., 2006; Shen et al., 2013). *In vitro* calibration uses heterologously expressed sensors in buffers of different pH. Such a calibration does not take into account the ionic strength and buffering capacity of the cell and thus can lead to substantial artifacts (Schulte et al., 2006). *In vivo* calibration uses sensor-expressing plant cells that are treated with an ionophore, such as nigericin, in presence of a solution with sufficient buffering capacity to clamp the pH of the cellular environment containing the sensor. This approach should be favored whenever possible, but is still limited for several reasons. First, there is no objective way to ensure that the equilibrium has reached between the bathing media and the cell compartment where the sensor is located. This is especially true when the sensor is in the lumen of intracellular structures, meaning that the ionophore has to be effective on two successive membranes. Secondly, the ionophore modifies the ion content of the cell and consequently the native buffering capacity and ionic strength. Such calibration issues may explain the ongoing debate on the feasibility of measuring absolute pH values with sensors *in vivo* (Schulte et al., 2006; Bizzarri et al., 2009).

## ANALYSIS OF EXOCYTOSIS EVENTS WITH pH SENSORS

Genetically encoded pH sensors can be used to investigate processes that give rise to, or rely on, changes in pH. During exocytosis, vesicle lumen undergoes a rapid alkalinization upon fusion to the plasma membrane. Since the pioneering study by Miesenböck et al. (1998), ecliptic pHluorin has been extensively used as an exocytosis indicator (Sankaranarayanan et al., 2000; Gandhi and Stevens, 2003; Taylor et al., 2011). For instance, in the synaptic cleft, Gandhi and Stevens (2003) were able to distinguish three types of vesicle behaviors at the plasma membrane. In another study, ecliptic pHluorin used to describe the dependency of vesicle fusion on actin filaments and cdc42, a protein controlling cell division (Alberts et al., 2006). Similar approaches would appear useful in the plant field. According to the classical view of ligand–receptor trafficking, a difference in pH is expected between the lumen of the *trans*-Golgi network (TGN) and the apoplastic face of the plasma membrane (Robinson et al., 2012). Hence, the luminal pH of exocytic vesicles is predicted to be different before and after fusion with the plasma membrane. In recent reports, TGN compartments have been shown to have the most acidic lumen of the secretory pathway, with a pH around 6 (Martinière et al., 2013; Shen et al., 2013; **Table 2**). On the other side, apoplastic pH is likely to be highly variable and dependent on tissue type and development stage (Gao et al., 2004; Staal et al., 2011; Geilfus and Mühling, 2012; Gjetting et al., 2012). Unfortunately, studies that concomitantly measure the pH in the TGN and in the apoplasm are still lacking.

**Table 2:** ummary of pH measurements in plant cells using pH sensors.

	Tobacco epidermis (Martinière et al., 2013)	*Arabidopsis* root tip (Martinière et al., 2013)	*Arabidopsis* protoplasts (Shen et al., 2013)
Cytosol	7.8 (pHluorin)	8 (pHluorin)	7.3 (PpHluorin)
ER	7.5 (pHluorin-hdel)	7.7 (pHluorin-hdel)	7.1 (PpHluorin-hdel)
*Cis*-Golgi			6.8 (ManI-PpHluorin)
*Trans*-Golgi	6.9 (ST-pHluorin)	6.8 (ST-pHluorin)	
TGN	6.1 (pHluorin-AtVSR2)	6 (pHluorin-AtVSR2)	6.2 (PpHluorin-AtVSR2 trunc)
PVC	6.8 (pHluorin-AtVSR2)		
Late PVC	7.1 (pHluorin-AtVSR2-IMAA)		
TGN plus recycling endosomes	6.5 (pHluorin-AtVSR2-YA)		6.3 (PpHluorin-truncAtVSR2-YA)
Vacuole	6 (BCECF-AM)	5.7 (BCECF-AM)	5.2 (Aleurain-PpHluorin)
Plastid stroma			7.2 (RecaA-PpHluorin)
Peroxisome			8.4 (PpHluorin-SLR)
Mitochondria			8.1 (mito-PpHluorin)
Nucleus			7.2 (NLS-PpHluorin)

*The average pH is indicated for each subcellular compartment the pH sensor used for the measurement being indicated between parenthesis. ST, sialyltransferase; ManI, mannosidase 1; AtVSR2, At2g14720; trunc, truncated AtVSR2 with no luminal domain; BCECF-AM, 2 ′,7 ′-Bis(2-carboxyethyl)-5(6)-carboxyfluorescein; tetrakis(acetoxymethyl) ester mito, transit peptide of the β-subunit of *Np*F1-ATP synthase; NLS, nuclear localization signal.*

## pH WITHIN THE APOPLAST

In plants, the plasma membrane is energized by P-type proton pump ATPases. Direct evidence has been obtained that proton diffusion within the apoplast away from the membrane can be rate-limiting when compared to proton excretion by the proton pumps. Using weak acid influx for assaying pH, it has been shown that the local pH in the unstirred aqueous layers next to the plasmalemma can be much lower than that of the external bathing solution, by up to 2 pH units under conditions that maximize the rate of net proton excretion (Sentenac and Grignon, 1987; Grignon and Sentenac, 1991). A few studies on the dependency of membrane transport in roots on external pH have quantitatively assessed this steady state pH shift at the membrane surface due to proton excretion by using weak acid influx assays under the same conditions as those used for the transport measurements (Thibaud et al., 1998). Clearly, the results indicated that this shift in pH strongly contributed to energization of the cell membrane and had to be taken into account to explain, for instance, kinetic and thermodynamic features of nitrate, phosphate, or potassium transport (Thibaud et al., 1988), or the effect of ${HCO}_{3}^{-}$, a buffer that can be naturally present in the external solution (in calcareous soils), on the transport rate of these nutriments (Toulon et al., 1989) or on iron reduction at the cell surface (Toulon et al., 1992).

Different methods have been developed to estimate local pH values, using proton-selective micro-electrodes (Felle, 1998) or impermeable pH-sensitive dyes (Taylor et al., 1996; Monshausen et al., 2009, 2011), or collecting apoplasmic fluids (Mühling and Sattelmacher, 1995). However, such approaches provide estimates of the pH conditions at the organ surface or, at best, of the mean pH within the apoplast, and not of the local pH in close vicinity of the membrane, which is the actual pH sensed by the ion transporters. Another source of difficulties is the possibility of heterogeneities within the apoplast. For example, in the root cortex and for the inner cortical cells, the diffusion barrier that slows proton migration toward the external solution comprises the entire apoplastic continuum, from cortical cells surrounding the endodermis to the root epidermis surface. Furthermore, longitudinal heterogeneities in surface pH have been observed between the root apex, the elongation region and the fully differentiated zone, due to differences in the rate and direction of proton transport. The physiological consequences and roles of such radial and longitudinal differences, in terms of membrane energization and transport activity, have been poorly investigated. Clearly, genetically encoded pH sensors open novel perspectives in this domain of the root structure–function relationship. In this respect, Moseyko and Feldman (2001) and Gao et al. (2004) have developed pioneering approaches. For example, large pH variations were observed in response to salt stress. When challenged with 100 mM NaCl, root cells displayed a more alkaline apoplasm and a more acidic cytosol. These changes in pH might result from specific activation of H^+^:Na^+^ antiport activity mediated by the plasma membrane Salt-Overly-Sensitive1 (SOS1) antiporter in response to salinity constraint (Quintero et al., 2011). Such analyses clearly indicate that expression of pH sensor proteins in mutant plants could allow rapid progress in investigating the root structure–function relationship and its role in membrane energization, ion transport and plant adaptation to environmental stresses.

## CELL WALL pH AND GROWTH

Cell capacity to control the apoplastic pH plays a major role also in plant growth and development. Indeed, according to the acid growth theory, cell wall “loosening” favored by low pH allows cell elongation (Rayle and Cleland, 1992). In a typical sequence of events, auxin stimulates proton secretion by plasmalemma proton pump ATPases, resulting in cell wall acidification, causing activation of expansins, which loosen cellulose microfibrils. As a result, cell walls become more extensible favoring turgor-driven cell elongation. In support to the acid growth theory, the elongation zone of the root generally displays a more acidic apoplasm than the fully differentiated zone (Staal et al., 2011). Intriguingly, a close-up analysis using a pH sensor reveals highly dynamic fluctuations of root surface pH with alkalinization and acidification periods and alkalinization bursts along the root longitudinal axis or alternating between opposing flanks (Monshausen et al., 2011). Such results indicate that a net influx of protons can occur in epidermal cells, as previously reported in other studies (O’Neill and Scott, 1983). Intriguingly, a recent report shows that treatment of root tips with indole-3-acetic acid (IAA) induces an increase in pH by about 0.5–0.8 pH units (Gjetting et al., 2012), which might be related to the periodical alkalinization previously reported in root tips (Monshausen et al., 2011).

## pH MAPPING OF ORGANELLES AND PROTEIN TARGETING

Taking advantage of growing knowledge in protein targeting mechanisms, appropriate signal sequences can be fused to the coding sequence of the pH sensor so that this is targeted later to various cell membranes or compartments. Using such an approach, pH in various intracellular compartments was measured *in vivo* in mammalian and, more recently, in *Arabidopsis* protoplasts (Shen et al., 2013), tobacco leaf epidermal cells or *Arabidopsis* root tip cells (Martinière et al., 2013). These studies directly demonstrate a gradual acidification of 1.5–2 pH units in the lumen of the endomembrane system with ER displaying the most alkaline pH and vacuole the most acidic one (see **Table 2**). Interestingly, some pH differences exist for a given compartment between plant species (cytosol, endoplasmic reticulum and vacuole; **Table 2**). These variations could be the result of biological variation between the systems tested (intact cells versus protoplast) or could be due to differences in growing condition. For instance, pH homeostasis is known to be sensitive to light intensity (Martinière et al., 2013). Most compartment markers cycle at least between two subcellular localizations and there is still no way to target a sensor solely to either the lumen of the TGN, the prevacuolar compartment (PVC) or the late PVC. Fusion of pH sensors to the vacuolar sorting receptor (VSR) mutants Y165A and IMAA allowed to partially solve such localization issue, the former mutant being preferentially retained in the TGN and the latter localized in the LPVC. This allowed differentiation between TGN and the PVC and getting pH estimates for these two compartments (Martinière et al., 2013; Shen et al., 2013). However, it should be noted that, in addition to TGN, the Y165A VSR mutant is very likely to also label recycling endosomes and plasma membranes (daSilva et al., 2006; Saint-Jean et al., 2010). Furthermore, accumulation of the IMAA mutant in the LPVC is only transient (Saint-Jean et al., 2010).

In average, the population of VSR labeled compartments has a pH intermediate between the *trans-*Golgi and the vacuole in all the systems tested. But surprisingly, the TGN is actually more acidic, with a pH 6, than the PVC (pH 6.8) or even the late PVC (pH 7.1 see **Table 2**; Martinière et al., 2013). This result fits well with the localization of V-type ATPase involved in pumping protons. Indeed, VHA-a1 protein is preferentially accumulated in the TGN (Dettmer et al., 2006) and is not detected in the multivesicular bodies (MVB; Robinson et al., 2012) or PVC. Accordingly to the proton leak model, while V-type ATPase acidifies a compartment, antiporters could be involved in proton extrusion (Demaurex, 2002). For instance, NHX5 and NHX6 Na^+^/H^+^ antiports have been proposed to be involved in such a mechanism in plant endosomes (Bassil et al., 2011). Therefore, the relative amount of VHA-a1 and proton antiporters such as NHX5/6, in compartment on the way to the vacuole could have a preponderant role in proton homeostasis and substantial consequence in terms of protein sorting. In support to this hypothesis, is the mistargeting of a soluble vacuolar marker in *nhx5 nhx6* double knockout (Bassil et al., 2011) and the fact that the pH measured in the TGN and the PVC fits well with the distribution of VHA-a1 and NHX5/6 in tobacco leaf epidermal cells (Martinière et al., 2013).

The fact that the PVC is more alkaline than TGN is surprising and in contradiction with the VSR-mediated vacuolar sorting model where acidification in the PVC is thought to be responsible for a release of the ligand by the receptor (Kirsch et al., 1994; Ahmed et al., 2000; Shimada et al., 2003). In fact, the *in vitro* binding curve between VSR and the ligand is bell shaped with an optimum pH at 6 (Kirsch et al., 1994). This value corresponds to the pH measured *in vivo* in the TGN (Martinière et al., 2013) suggesting that binding of VSR to its ligand would be optimal in the TGN but significantly lower in more acidic and alkaline compartments. The use of ratiometric sensors to measure the endomembrane luminal pH therefore suggests that alkalinization rather than acidification would trigger ligand release from the receptor VSR in the PVC (Martinière et al., 2013). Of course this model does not take into account other environmental parameters in TGN and PVC such as the calcium concentration that is also known to regulate ligand binding for a least some members of the VSR family (Watanabe et al., 2002, 2004). Finally, it is also important to point out that the pH reported for the PVC or the TGN is an average from a large population of independent organelles with some variation of pH values. This difference is either due to technical limitations in imaging or may reflect pH variability between compartments of same identity, e.g., TGN or PVC. A model for VSR-mediated transport is still a matter of debate (Robinson and Pimpl, 2013).

It is likely that pH ratiometric sensors will open new perspectives in our understanding of the mobility, functional heterogeneity and remodeling capacities of organelles. For instance, the TGN has been assumed to undergo maturation as its morphology changes over time (Scheuring et al., 2011). Using pH sensors along with dynamic studies could provide evidence for these maturation events.

## CONCLUDING REMARKS

Genetically encoded pH sensors have opened avenues for cell biology. They will provide valuable functional data with respect to cell compartmentation, protein trafficking, acidic growth, or ion diffusion within plant tissue. One of the future challenges will be to improve the definition of pH information provided by the sensor as cell nano-environments are anticipated. For instance, protein and lipid composition of membranes have a substantial effect on proton diffusion. As well, it is reasonable to anticipate a higher proton concentration in the vicinity of ATPase pumps and therefore heterogeneity on membrane surfaces with respect to pH.

## Conflict of Interest Statement

The authors declare that the research was conducted in the absence of any commercial or financial relationships that could be construed as a potential conflict of interest.
